# A comparison of the disease occurrence of cerebrovascular diseases, diabetes mellitus, hypertensive diseases, and ischaemic heart diseases among hospitalized older adults in Thailand

**DOI:** 10.1038/s41598-023-49274-z

**Published:** 2024-01-02

**Authors:** Passakorn Suanrueang

**Affiliations:** Department of Health Education and Behavioral Sciences, Faculty of Public Health, Mahidol University, Bangkok, Thailand

**Keywords:** Diseases, Health care

## Abstract

This observational research analyzed public hospital data from the Thailand Ministry of Public Health website to investigate gender differences in four categories of non-communicable diseases (NCDs) affecting hospitalized senior Thai populations for 12 years. This study aimed to determine the cumulative effects and analyze the odds ratio (OR) according to ICD-10 cause categories for the data from 2010 to 2021, accounting for 1,327,093 cases in 2010 and 2,275,936 cases in 2021. The findings revealed statistically significant gender differences in four categories of NCDs. Men were found to be more likely than women to have two types of NCDs, as measured by the OR (95%CI): cerebrovascular diseases (OR 1.34–1.47, 95%CI 1.32–1.48), and ischaemic heart disease (OR 1.24–1.63, 95%CI 1.23–1.64). Conversely, diabetes mellitus (OR 0.64–0.84, 95%CI 0.63–0.85) and hypertensive disorders (OR 0.82–0.95, 95%CI 0.81–0.97) were discovered to have a lower likelihood of ratios related in men compared to women. However, the trend of all four NCDs in men has significantly increased every year: cerebrovascular diseases = 0.0093 year(s) + 1.3391, (R^2^ 0.82, *p*-value 0.001); diabetes mellitus = 0.0171 year(s) + 0.6143, (R^2^ 0.97, *p*-value 0.001); hypertension = 0.0125 year(s) + 0.8091, (R^2^ 0.96, *p*-value 0.001); and ischaemic heart disease = 0.0345 year(s) + 1.1884, (R^2^ 0.99, *p*-value 0.001).

Gender, a crucial biological factor, contributes to variations in the prevalence of illness. As such, it is essential to prioritize the disease risk occurrence and preventive care for men and women separately, with a focus on implementing more detailed screening and detection strategies, as well as tailored interventions.

## Introduction

As previously established, non-communicable diseases (NCDs) are significant contributors to premature mortality worldwide, with approximately 86% of premature mortality in low and middle-income countries. Each year, around 17 million individuals who died before the age of 70 years being affected by these diseases^1^. Over the last decade, NCDs have emerged as a major global public health concern. NCDs, also known as chronic diseases, are characterized by their prolonged duration and persistence. The World Health Organization (WHO) recognizes five main categories of NCDs: cardiovascular diseases, cerebrovascular diseases, neoplasms, diabetes mellitus, and chronic obstructive pulmonary diseases^1^. These diseases are responsible for a significant portion of global morbidity and mortality and have significant economic and social implications^2^. This has led to increased attention on the prevention and management of NCDs by global health organizations, governments, and healthcare professionals^3^.

Thailand, like many other countries, is experiencing a demographic shift with a growing proportion of older adults in its population^4^. In other words, Thailand has become an aging society, indicating a lower birth rate and a higher proportion of senior people. In this population structure shift, we are facing the big challenges of age-related health issues or increasing ages that come along with health conditions. This phenomenal, no doubt, impact health-related quality of life of senior populations^5^.

The increasing burden of NCDs among this age group calls for a better understanding of disease patterns, risk factors, occurrences, or determinants of health related to the development of diseases to inform targeted healthcare interventions and resource allocation. One of the social determinants of health that needs to be put the approaching healthcare administration into action is gender, a biological factor^6,7^. Several previous research has indicated the differentiation between males and females in disease incidence and prevalence.

Cerebrovascular diseases are a subset of NCDs, and although men and women may get a chance of developing them equally, some studies have reported that men are more likely to suffer and are at higher risk of intracerebral hemorrhage (ICH) compared to women^8–11^. Stroke is also a subgroup of cerebrovascular diseases^12^. Men have been reported to have a higher occurrence of stroke, especially subarachnoid hemorrhages (SAHs) with an early onset of stroke, than women^13^. However, women are more likely to suffer from cardioembolic strokes, which are widespread in women^13^.

Ischemic heart disease (IHD) is one of the significant diseases that affects the proportion of mortality worldwide. This disease refers to two clinical presentations, namely coronary artery disease (CAD) and atherosclerotic cardiovascular disease (ACD)^14^. An epidemiological study^14^ in a global dataset (1990–2017) indicates that ischemic heart disease was found to have a higher proportion in men with increasing ages. Similarly, other three studies^15,16^, found the same results that the incidence of ischemic heart disease or related conditions as cardiovascular diseases^17^ demonstrated a noteworthy differentiation between genders, with men experiencing significantly higher rates than women.

Diabetes mellitus is a subset of endocrine diseases involving high blood sugar levels. Women have commonly reported a trending higher risk of this condition than men, especially diabetes type II^18^. Moreover, there is a considerable connection between physiological health conditions and mental and psychological consequences. Women with major depressive disorders (MDD), for example, have been found to have a strong linkage with diabetes mellitus, which is a factor contributing to MDD^19^. In addition, diabetes mellitus is an important factor in stimulating several other health outcomes, such as muscle discomfort, urinary signs, neurological signs, and skin-related indications^20^. Comparing among ages, men have a higher likelihood of developing diabetes at a younger age, in particular individuals who have a lower body mass index (BMI). On the other hand, women were found a higher experience a significant rise in the risk of cardiovascular diseases associated with diabetes following menopause^21^.

Hypertensive disease is the primary contributor to the risk of cardiovascular disease. Hypertension or high blood pressure was reported prevalent in different genders. Some studies reported a high prevalence of hypertension in women^22^, and this condition matches with increasing age^23^. In the case of men, a study indicated that young men were reported to have a higher prevalence of high blood pressure than same-age women^23^.

From the above evidence, it is obvious that gender is one of the notable health determinants. Therefore, recognizing potential gender differences in disease occurrence is crucial for developing gender-specific preventive strategies and optimizing healthcare delivery. In Thailand, a significant proportion of the elderly population is affected by non-communicable diseases and requires prolonged treatment in hospitals. However, there is limited evidence to support research on the long-term comparison of disease risk occurrences, particularly over ten years between genders. Therefore, this study aimed to analyze the differences in the rates of the four significant NCDs between genders and identify trends and patterns in the likelihood of these NCDs among senior males and females. This information is crucial for understanding historical phenomena and predicting future trends in gender differences in NCDs among senior populations in Thailand.

## Methods

### Study design

This observational study was designed to investigate the statistical analysis of non-communicable disease rates by gender, examine trends, and calculate the probability ratio of non-communicable diseases occurring between genders. The primary focus of this current study is on exploring the diseases as potential complications of chronic illnesses, with a specific emphasis on cerebrovascular diseases, diabetes mellitus, hypertensive diseases, and ischemic heart diseases leading causes of chronic illnesses in older adults hospitalized in Thailand.

### Source of data

Data were analyzed to investigate the ratio of these four NCDs per 100,000 populations by specific disorders and sexes and the trend over a 12-year period. The study used data on both the likelihood of the occurrence of the four NCDs and the likelihood of it not occurring as sources of information.

### Methods of data collection

This quantitative study obtained secondary data published on the official website of the Ministry of Public Health, Thailand. The data were available in the form of a report (provided only in Thai language versions) by the Strategy and Planning Division of the Office of the Permanent Secretary, the Ministry of Public Health. These reports included information on the number and prevalence rates of outpatients and inpatients by disease subgroups identified with disease titles and diagnostic codes based on the International Statistical Classification of Diseases and Related Health Problems (ICD-10). The data was gathered by selecting from the handling of diagnostic Data.

### The probability of diseases

#### Number of patients by gender and year

Data for this study were obtained from the website of the Ministry of Public Health in Thailand. The number and prevalence rate of four NCDs among elderly patients (aged 60 years and over) who were hospitalized in public hospitals in Thailand, were reported by sex and year, covering the period from 2010 to 2021. The data for each year included all disease diagnoses. The prevalence rates per 100,000 populations of four NCDs were calculated and reported by the Health Data Center (HDC), the Ministry of Public Health, Thailand.

$$\frac{{{\text{Number of hopitalized patients for one year }}\; \times \;100,000}}{{{\text{Mid}} - {\text{year population in the same year}}}}$$

### Handling of diagnostic data

This research concentrated on NCDs, utilizing the ICD-10, and was based on the disease subgroups classified by the Health Data Centre, Ministry of Public Health of Thailand. There were four subgroups by disease titles and diagnostic codes; (1) cerebrovascular diseases (I60-I69) which included intracranial haemorrhage (I60-I62), cerebral infarction (I63), stroke, not specified as haemorrhage or infarction (I64), and other cerebrovascular diseases (I65-I69), (2) diabetes mellitus (E10-E14), (3) hypertensive diseases (I10-I15) which included essential (primary) hypertension (I10), and other hypertensive diseases (I11-I15), and (4) ischaemic heart diseases (I20-I25) which included acute myocardial infarction (I21-I22), and other ischaemic heart diseases (I20, I23-I25).

### Statistical analysis

The study presented the prevalence rate of hospitalized patients who are aged 60 years and over with NCDs and investigated gender differences. The NCDs were divided into four categories. This study focused on the odds ratio (OR) for each disease as defined by ICD-10 criteria in the four categories of diseases, to examine the association between genders and each group of diseases and compare the probabilities of occurrence by gender.

Based on previous findings, it has been indicated that men are more likely to be at risk of two health conditions: cerebrovascular diseases and ischemic heart diseases. Therefore, it was hypothesized that these conditions have a higher prevalence among men. The OR was calculated as the disease occurring of cerebrovascular diseases and ischemic heart diseases in males to the disease occurring in females and the total population. It was used as female as a baseline measure of the comparison of the ratio between gender and the disease. The level of uncertainty surrounding this measure was indicated by the 95% confidence interval (CI) by using SPSS, the statistical package, for data entry and analysis.

**Table:** 

A 2 × 2 contingency table for cerebrovascular diseases			A 2 × 2 contingency table for ischemic heart diseases		
	Cerebrovascular diseases (Event)	No Cerebrovascular diseases (Non-event)		Ischemic heart diseases (Event)	No ischemic heart diseases (Non-event)
Male	a	b	Male	a	b
Female	c	d	Female	c	d
**Note;** “a” is the number of hospitalized males with cerebrovascular diseases “b” is the number of males without cerebrovascular diseases. (Overall target population—number of hospitalized males with cerebrovascular diseases) “c” is the number of hospitalized females with cerebrovascular diseases “d” is the number of females without cerebrovascular diseases. (Overall target population—number of hospitalized females with cerebrovascular diseases) OR = (a/b)/(c/d)			**Note;** “a” is the number of hospitalized males with ischemic heart diseases “b” is the number of males without ischemic heart diseases. (Overall target population—number of hospitalized males with ischemic heart diseases) “c” is the number of hospitalized females with ischemic heart diseases “d” is the number of females without ischemic heart diseases. (Overall target population—number of hospitalized females with ischemic heart diseases) OR = (a/b)/(c/d)		

On the other hand, previous studies indicated that females are more at risk for diabetes mellitus, and hypertensive diseases than men. Therefore, it was hypothesized that these conditions have a higher prevalence among women. The OR was calculated as the disease occurring of diabetes mellitus, and hypertensive disorders in females to the disease occurring in males and the total population. It was used as males as a baseline measure of the comparison of the ratio between genders and the diseases. The level of uncertainty surrounding this measure was indicated by the 95% confidence interval (CI).

**Table:** 

A 2 × 2 contingency table for diabetes mellitus			A 2 × 2 contingency table for hypertensive disorder		
	Diabetes mellitus (Event)	No diabetes mellitus (Non-event)		Hypertensive disorders (Event)	No hypertensive disorders (Non-event)
Female	a	b	Female	a	b
Male	c	d	Male	c	d
**Note;** “a” is the number of hospitalized females with diabetes mellitus “b” is the number of females without diabetes mellitus. (Overall target population—number of hospitalized females with diabetes mellitus) “c” is the number of hospitalized males with diabetes mellitus “d” is the number of males without diabetes mellitus. (Overall target population—number of hospitalized males with diabetes mellitus) OR = (a/b)/(c/d)			**Note;** “a” is the number of hospitalized females with hypertensive disorders “b” is the number of females without hypertensive disorders. (Overall target population—number of hospitalized females with hypertensive disorders) “c” is the number of hospitalized males with hypertensive disorders “d” is the number of males without hypertensive disorders. (Overall target population—number of hospitalized males with hypertensive disorders) OR = (a/b)/(c/d)		

Furthermore, odd ratio trends over a 12-year period were calculated and predicted the gender dissimilarity in four NCD categories by testing the ability of time to predict increased odds ratios by using simple linear regression with a presented R-square, ANOVA test, and corresponding values.

### Ethics approval and consent to participate

This research analyzed secondary data that was accessible to the public from the Thailand Ministry of Public Health’s website, such as data on gender and year-specific numbers and rates of occurrence. The data was anonymous and did not include personal information, therefore, institutional review board approval was not required.

## Results

The prevalence rates of non-communicable diseases among older males and females are presented in Table 1. On the one hand, males had a higher prevalence rate of cerebrovascular diseases and ischaemic heart disease than females. The prevalence rate (per 100,000 populations) for cerebrovascular diseases in males was from 2169.00 in 2010 to 2503.56 in 2021, while the prevalence rate for females was between 1630.80 in 2010 and 1717.05 in 2021. Similarly, the male prevalence rates for ischaemic heart disease were 2885.81 in 2010, hit a peak at 3026.51 in 2015, and fluctuated to 2498.01 in the last period of the study, while the female rates were lower than that of male rates, beginning with 2344.69 in 2010 and continuously declining to 1545.91 in 2021, which is lower than males at around 800 cases per 100,000 population (Table 1).

**Table 1:** The prevalence rates of four NCDs (cerebrovascular disease, diabetes mellitus, hypertensive diseases, and ischaemic heart disease) per 100,000 population by gender among hospitalized older patients (60 years and above) in public hospitals from 2010–2021.

	ICD-10 diagnosis descriptions							
	Acute myocardial infarction and Other ischaemic heart diseases		Cerebrovascular diseases		Diabetes mellitus		Essential (primary) hypertension and other hypertensive diseases	
Year	Male	Female	Male	Female	Male	Female	Male	Female
2010	2885.81	2344.69	2169.00	1630.80	3959.68	6058.97	7408.82	8888.30
2011	2944.43	2331.63	2256.96	1671.22	4029.19	5979.25	7749.87	9112.97
2012	2943.32	2291.12	2337.00	1713.20	4240.29	6205.11	8207.12	9558.15
2013	2862.99	2175.23	2318.85	1680.91	4228.55	6065.26	8176.83	9334.27
2014	2589.83	1948.78	2125.85	1516.59	3910.92	5563.25	7585.77	8602.31
2015	3026.51	2199.14	2472.83	1781.12	4561.55	6316.33	8875.03	9981.96
2016	2963.38	2073.17	2450.63	1799.14	4677.94	6390.03	9149.56	10,131.45
2017	3013.11	2039.68	2488.64	1790.78	4787.75	6359.88	9260.14	10,141.29
2018	2890.23	1953.82	2587.85	1837.61	5050.85	6508.60	9694.41	10,424.93
2019	2812.44	1859.00	2594.07	1836.93	5060.40	6441.48	9660.80	10,351.11
2020	2811.42	1797.56	2654.67	1856.79	5129.42	6275.71	9766.18	10,032.67
2021	2498.01	1545.91	2503.56	1717.05	4843.07	5681.91	9009.87	9438.16

On the other hand, two non-communicable disease categories, namely diabetes mellitus and hypertensive disorders, had been found to have a higher prevalence rate in females overall after 12 years of observation. The prevalence rate for diabetes mellitus in females was higher than approximately 6000 per 100,000 population, while the prevalence rate in males was between 4000 and 5100 per 100,000 population. Likewise, the prevalence rate of hypertensive diseases in females was higher than in males, with approximately 1480 cases per 100,000 people reported in females in 2010. However, the prevalence rate in the last period of the study in 2021 was close to the same. The cases in females were higher, with approximately 430 cases per 100,000 population than those in males (Table 1).

**Figure 1:**
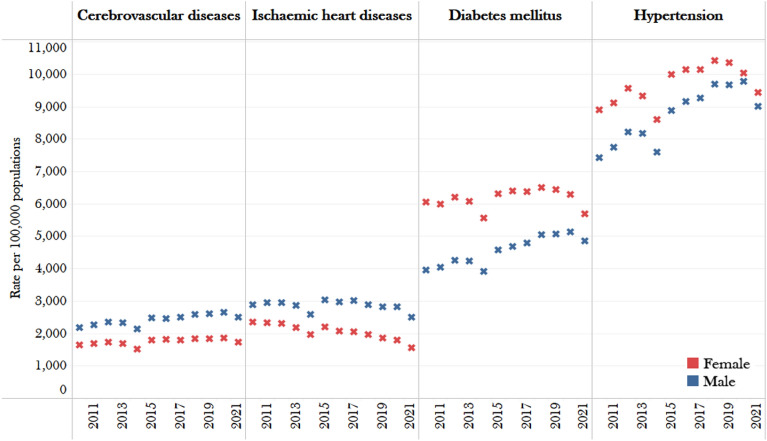
Rate of four diseases (cerebrovascular diseases, ischaemic heart diseases, diabetes mellitus, and hypertensive diseases) per 100,000 population by sex, 2010 and 2021.

Figure 1 illustrates the gender differences in non-communicable diseases by presenting the rate and trend over a 12-year period for four groups of non-communicable diseases per 100,000 population. It is evident from the figure that cerebrovascular diseases (close to 2100–2650 per 100,000 populations) and ischemic heart diseases (approximately 2500–3000 per 100,000 populations) had a higher prevalence rate in males over a 12-year period. In contrast, females were more likely to experience diabetes mellitus (around 5560–6500 per 100,000 populations) and hypertension and related disorders (approximately 8600–10,400 per 100,000 populations).

**Figure 2:**
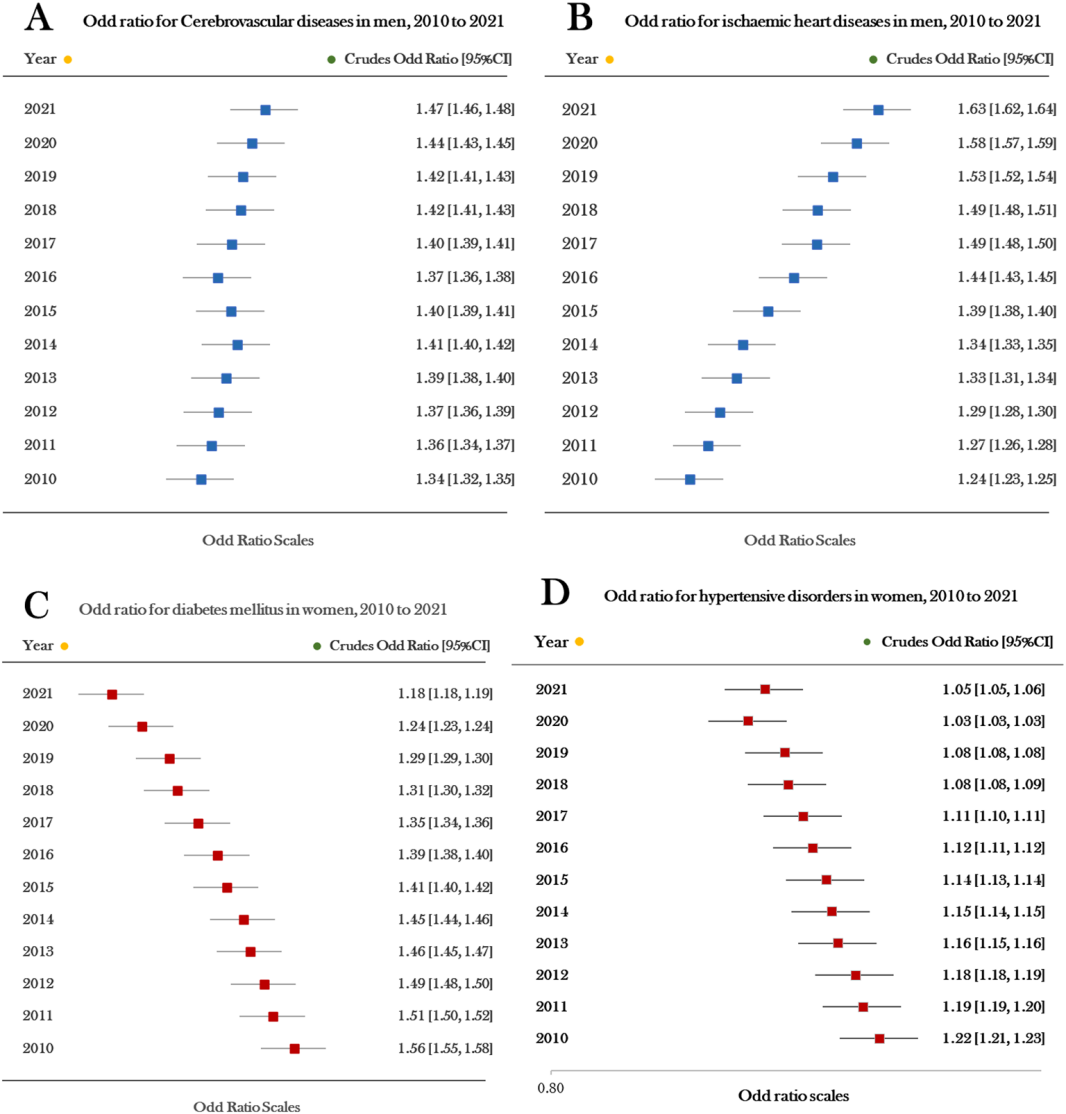
The odd ratio for four non-communicable diseases in men and women (greater than 1) between 2007 and 2019. Horizontal lines indicate corresponding 95% confidence intervals around odd ratio: (**A**) cerebrovascular diseases in men; (**B**) ischaemic heart diseases in men; (**C**) diabetes mellitus in women; (**D**) hypertensive disorders in women.

Based on prior research, it has been observed that cerebrovascular diseases and ischaemic heart diseases are more prevalent in men, while diabetes mellitus and hypertensive disorders are more common in women. Therefore, in this analysis, cerebrovascular diseases and ischaemic heart diseases were used with females as the reference group, and diabetes mellitus and hypertensive disorders were compared with males as the reference group to analyze the crude odd ratio (COR). Trends in the COR for men and women with non-communicable diseases are presented in Fig. 2. Among men, two NCDs, namely cerebrovascular diseases and ischaemic heart diseases, displayed significantly higher COR values with increasing trends over a 12-year period of study. On the contrary, diabetes mellitus and hypertensive disorders were found to affect more women than men. However, the trends for these two NCDs in women show a decreasing pattern. For instance, the COR (95%CI) for diabetes mellitus in women was 1.56 (95%CI 1.55–1.58) in 2010, which decreased to 1.18 (95%CI 1.18–1.19) in 2021. Similarly, the COR for hypertensive disorders in women was 1.22 (95%CI 1.21–1.23) in 2010, but it decreased to 1.05 (95%CI 1.05–1.06) in 2021 (Fig. 2).

**Figure 3:**
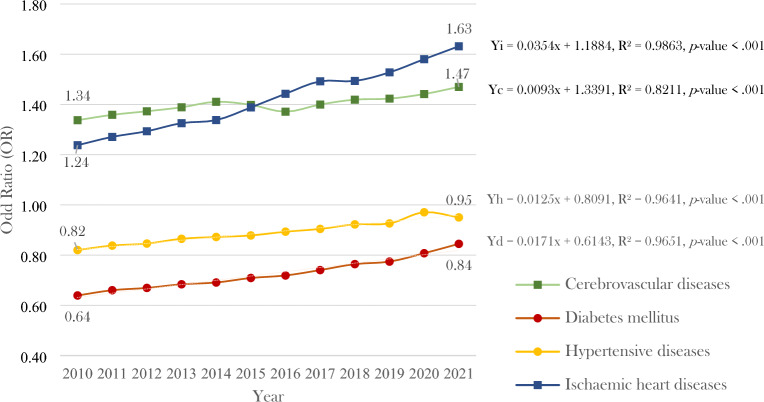
The odds ratios for males (females as a baseline, *p*-value < .001) were higher than 1 for the occurrence of four NCD categories for the 12 years, between 2010 and 2021. The four NCDs are specified in separate lines. (1) the blue line denotes ischaemic heart disease, (2) the green line denotes cerebrovascular disease, (3) the yellow line represents hypertensive disease, and (4) the red line represents diabetes mellitus.

The findings illustrated in Fig. 2 reveal that cerebrovascular diseases and ischaemic heart diseases have higher COR in males. However, diabetes mellitus and hypertensive disorders displayed higher COR in females, and the trend gradually decreased. In other words, these two disorders gradually increased in males. Therefore, it is interesting to explore the trend of these two health conditions (diabetes mellitus and hypertensive disorders) in men. This exploration assumes that the OR may change over time, under the condition that provided healthcare services and other relevant factors remain constant. The analysis in question focuses on a time span and investigates the trend after 12 years. Its purpose is to investigate the odds ratio (OR) for each disease in subsequent years (13th year and next) focusing on male health conditions. Females were used as a reference group for this analysis, and the results are presented in Fig. 3. Additionally, a prediction analysis was performed to test for an increasing trend over the years in terms of increased odds ratio, and the results are presented in Table 2.

**Table 2:** The correlation between time (years) and increased odds ratio of four NCDs in men and a simple regression equation with a model summary and an ANOVA test by four NCDs categories.

NCDs	n	Model summary				ANOVA test	Coefficients	
		R	R-square	Adjusted R-square	Standard Error	F (*p*-value)	Regression equation	T (*p*-value)
Cerebrovascular diseases	12	0.91	0.82	0.80	0.02	45.88 ***	Yc = 0.0093x + 1.3391	6.77***
Diabetes mellitus	12	0.98	0.97	0.96	0.01	276.54 ***	Yd = 0.0171x + 0.6143	16.63 ***
Hypertensive diseases	12	0.98	0.96	0.96	0.01	268.36 ***	Yh = 0.0125x + 0.8091	16.63 ***
Ischaemic heart diseases	12	0.98	0.99	0.98	0.02	721.16 ***	Yi = 0.0345x + 1.1884	16.38 ***

n = COR between 2010 and 2021 (12 years), Yc = cerebrovascular diseases, Yd = diabetes mellitus, Yh = hypertension, Yi = ischaemic heart diseases, x = time (year), *** *p*-value < 0.001.

A positive relationship between males and cerebrovascular diseases and ischemic heart diseases over a 12-year study period was revealed by an analysis of the odds ratio. The odds ratio for cerebrovascular diseases in males was higher than 1 (females as a baseline, *p*-value < 0.001), with a value of 1.34 (95%CI 1.32–1.35) in 2010 and increasing to a peak of 1.47 (95%CI 1.46–1.48) in 2021. Similarly, ischemic heart disease also showed an increasing trend over time in males, with an odds ratio of 1.24 (95%CI 1.23–1.25) in 2010 and 1.63 (95%CI 1.62–1.64) in 2021 (Fig. 3). In the case of diabetes mellitus and hypertensive disorders, the OR was lower than 1. However, the trends for these two disorders were gradually rising. For diabetes mellitus, it started at 0.64 (95%CI 0.63–0.64) in 2010 and increased to 0.84 (95%CI 0.84–0.85) in 2021. Likewise, for hypertensive disorders, it began at 0.82 (95%: 0.82–0.82) and advanced to 0.95 (95%: 0.95–0.95), which was close to 1. (Fig. 3).

Table 2 presents a significant positive correlation (*p*-value < 0.001) between time (years) and the odds ratio in all four NCDs in males. Increased times (years) in the equation can predict an increase in the odds ratio in all four NCDs with a correlation (R^2^) between 0.82 and 0.99.

## Discussion

### A higher probability of developing two NCDs in men

The findings from this current study found that compared to older females, older males aged 60 years and over have a higher probability of developing two NCDs, particularly, cerebrovascular diseases and ischaemic heart diseases over the study period.

#### Cerebrovascular diseases

The current study on cerebrovascular diseases included different types of conditions such as intracranial hemorrhage, cerebral infarction, stroke unspecified hemorrhage or infarction, and other cerebrovascular diseases. The findings of this study align with those of other studies. In particular, when looking at specific diseases within the broader category of cerebrovascular diseases, there is evidence to suggest that there are differences in incidence rates between genders. A higher incidence of cerebrovascular diseases was found in men; for example, a study of around 1,000 hospitalized patients in Singapore found that males had a higher incidence of intracerebral hemorrhage (ICH) and tended to develop it at an earlier age than females^10^. Similar findings in Taiwan indicate that males had a higher rate of ischemic stroke^24^. Moreover, stroke occurred more frequently in men than in women in China^25^. Men had a higher chance of encountering blood accumulation in the basal ganglia region and also had a higher risk of mortality within three months after suffering from an intracerebral hemorrhage^26^.

Possible contributing factors to the differences in the incidence rate of cerebrovascular diseases might be considered in a variety of dimensions, especially biological, and behavioral factors. Biological markers associated with the risks of cerebrovascular diseases include body mass index (BMI), blood pressure, and hyperlipidemia^27^. The BMI is one of the modifiable risk factors; in the case of those who have a BMI over 30, it increases the risk of cerebrovascular diseases by the relative risk (RR), accounting for 2.00 for total stroke, 1.95 for ischemic stroke, and 2.25 for hemorrhagic stroke^28^. Reduced hypertension can significantly minimize the prevalence and mortality of stroke, as revealed by evidence from a study in Japan^29^. The study indicated that by addressing normal to severe hypertension, the incidence of stroke can be prevented in 64% of men and 50% of women, while stroke-related deaths can be reduced by 67% in men and 29% in women^29^. Additionally, it has been determined that health risk behaviors, especially alcohol consumption^30–32^ and smoking^33–35^ are significant contributors to stroke, ischemic stroke, or cerebrovascular diseases. Compared to females, males are more likely to engage in these risky health behaviors, which increases their risk of developing cerebrovascular diseases^36^.

#### Ischaemic heart diseases

Ischaemic heart disease is a significant cause of males admitted to receiving prolonged treatment in hospitals. The present study found that males have 1.23 to 1.63 times more at risk of ischaemic heart diseases than females. A study in the UK found that the incidence of acute myocardial infarction was significantly higher in males, with a ratio of approximately three times more likely than females^37^. However, in the case of females who have underly health conditions or comorbidities, especially diabetes mellitus, hyperlipidemia, and metabolic syndrome, their risk of developing acute myocardial infarction will exceed that of males^38^.

The factors contributing to ischaemic heart diseases, such as blood pressure levels, were significantly correlated with mortality and the prevalence of cardiovascular diseases between genders. The results of a study in Japan indicated that if there were therapeutic interventions to control the levels of hypertension, it could reduce the rate of incidence and mortality from CVD^29^.

### A higher probability of developing two NCDs in women

In terms of diabetes mellitus and hypertensive diseases, females were found to have a higher number of hospitalized patients compared to males over 12 years. This finding is consistent with a study conducted in Korea, which reported a greater prevalence of diabetes among women once they reached the age of 65^39^.

#### Diabetes mellitus

One of the most common predictors of increased risk of diabetes in women is overweight and obesity, especially in those who have a BMI over 30^40^. It is also insufficiently active in physical activities and has an unhealthy diet^40^. Furthermore, there are two significant factors in the biological process or metabolic abnormalities that stimulate diabetes mellitus, in particular diabetes type II. It included pancreatic β-cells that exhibit impaired insulin secretion and insulin-sensitive tissues that fail to respond adequately to insulin^41^. Women were indicated to have higher insulin resistance than men, especially those who have lower physical activities^42^. Furthermore, compared to men, women reported a greater probability of experiencing an increase in postprandial insulin and C-peptide concentrations following a meal^42^. Another evidence of gender differences in diabetes is the differentiation of pre-diabetic clinical dispositions. Men are more likely to report higher levels of impaired fasting glucose (IFG), whereas women are more prone to encounter impaired glucose tolerance (IGT)^42^. One of the supporting scientific results that demonstrate the lower level of IFG in women is the activity of gonadal hormones, especially estrogen, which has the potential to minimize fasting glucose. However, there is an argument that women have a higher possibility of IGT, which may be demonstrated by the different links to lifestyles and physical exercises in women^43^. The solution that was suggested to delay diabetes such as walking. Taking a walk two or three hours per week was recommended as a good solution to diminish diabetes type II. One of the benefits of walking is that it has the potential to induce skeletal muscle cell activities to provoke increasing glucose absorption in the blood flow from plasma into the muscle^41^.

#### Hypertensive diseases

The analysis results of the present study indicate that elderly women have a higher prevalence of hospitalized patients with hypertensive diseases compared to older men. This finding aligns with previous studies in American women^44^, and French women^22^ that reported a high prevalence of hypertension, or high blood pressure. Similarly, a previous analysis conducted in Korea on nationwide cases of ischemic stroke demonstrated that men had a higher prevalence of hypertension and hyperlipidemia until middle age, but beyond that age, women became more susceptible to these conditions^23,39^.

Gender differences in hypertensive diseases can be affected by age and hormonal changes, particularly after menopause. Compared to men, high blood pressure in women will gradually arise after menopause, and women aged 65 and over will have a higher possibility of developing hypertension than men^45^. After menopause, women may experience changes in hormonal levels, particularly a decrease in estrogen. Estrogen is thought to have a protective effect on the cardiovascular system, including helping to keep blood vessels flexible and promoting better blood flow. As estrogen levels decline during menopause, there may be an increased risk of developing hypertension in some women^45^. In addition to that, many factors contribute to the higher likelihood ratios of hypertensive diseases in women than men. Scientific and empirical evidence highlights the significance of sex hormones, the autonomic nervous system, the renin–angiotensin–aldosterone system, and arterial stiffness in the pathogenesis of persistently elevated blood pressure in women^46^.

Even though the analysis of the odds ratio revealed that the possibility of the risk of developing these NCDs (diabetes mellitus and hypertensive diseases) in males is less than 1, it tends to gradually increase over time with the number of populations. It suggests that in the coming years, there may be a prediction of similar numbers of elderly male and female patients hospitalized for these NCDs. This highlights the importance of gender-specific screening and targeted interventions aimed at addressing the specific needs of the senior male and female populations to prevent and control the rising trend of these diseases in both genders.

To conduct research in the long run and investigate with comparison of disease risk occurrences is important, which can help to fully understand the influences and dynamic of the development of NCDs on the senior population and other aged groups who are at risk of these diseases in Thailand. This would involve tracking the incidence and prevalence of NCDs in groups of senior populations and other vulnerable groups over a long-term period and comparing the results to those from previous years. The result from the research is able to provide valuable insights into the current phenomenon and inform predictions for future trends for policymakers or healthcare professionals to tailor the screening, diagnosis, and therapeutic interventions to align with the changing modern society.

### Implication and academic suggestions

The evidence from the current study highlights gender differentiation in non-communicable diseases and the increasing risk of certain NCDs over time. Many dimensions of implementation suggestions should be given importance, particularly preventive healthcare and wellness. For example:

Gender-specific health promotion campaigns should be developed and implemented by emphasizing lifestyle modifications, accessing early detection or disease risk screenings, and therapeutic options. Based on the current findings, senior males, who are at risk, should gain more awareness and preventive healthcare programs for cerebrovascular and ischaemic heart diseases. Senior females, on the other hand, should be more taken on board for diabetes mellitus and hypertensive disorders.

Promoting healthy lifestyles is still substantial. Interventions may be planned with consideration of tailoring variations in NCD prevalence between genders. Interventions, specifically health education, awareness initiatives, encouragement of self-efficacy, self-regulation, and so forth, involve healthy lifestyle choices based on socioeconomic status.

Public awareness and self-care should be launched as a foundation, especially the risk factors, the development of disease, the importance of engaging in self-care practices, and seeking healthcare services, by creating a public health awareness campaign. It is, however, considering the background knowledge of each individual, and may apply the KAP or social support theory to each group of people. Additionally, multidisciplinary approaches should be encouraged through sustainable collaborations with a variety of sectors and stakeholders, especially to create comprehensive programs targeted at the root causes of NCDs.

### Academic suggestions

Gender-specific variations are fascinating topics that have a substantial impact on determining health through genes and biology, ultimately affecting population health. Although this determinant of health is an unchangeable individual characteristic, we can delay the onset or contribution of diseases by understanding modifiable individual characteristics and factors surrounding them. Consequently, future studies should prioritize investigating factors linked to NCD prevalence via conducting in-depth research to find out the underlying causes of gender-based differences. Additionally, successful interventions should be developed and conducted research, especially the potential strategies that should be promoted as a starting point to encourage and empower people to improve self-efficacy, self-regulation, and so forth to increase preventive healthcare and wellness, and behavioral modification to be healthy and well-being.

## Conclusions

The present results highlight gender differentiation in four non-communicable diseases and present the predictive model (under the assumption that healthcare services and other relevant factors remain unchanged or consistent) that demonstrates the relationship between increasing time and disease occurrences (by odd ratios) over time by regression with a model summary, a correlation coefficient, and the results of an ANOVA test.

Non-communicable diseases, specifically cardiovascular disease, cerebrovascular disease, diabetes mellitus, hypertension, and related disorders, are significant global public health concerns. According to a recent study of historical data collected over 12 years, senior males in Thailand are more likely to develop two noncommunicable diseases: cerebrovascular diseases and ischaemic heart diseases, both of which require long-term pharmacotherapeutic treatments in hospitals, as well as this need high expenditure in medical care.

Although diabetes mellitus and hypertension were found to be more common in older females, males also have an increased chance of developing these health conditions, according to the current findings, which show an increasing trend in the odds ratio that increases year by year. Therefore, effective strategies for health promotion and prevention, and healthcare management of NCDs, are essential for addressing this global public health concern. These are commonly suggested and include promoting healthy lifestyles such as regular physical activity, a healthy diet, stopping smoking and tobacco use, and alcohol consumption, with strongly induced health behavior modification. As well as providing access to screening, early detection, and treatment services with align with changing modern society. Additionally, a multidisciplinary approach involving multiple sectors, including health education and strategies used to cope with chronic diseases, is necessary to effectively address the social determinants of NCDs and reduce health disparities. Importantly, gender-specific interventions need to be considered, which also requires a comprehensive and multidisciplinary approach.

Furthermore, one of the major challenges in healthcare management is promoting public awareness and self-care practices regarding health among individuals and encouraging them to engage in such practices as fundamental.

### Limitation

This study aimed to determine the magnitude of the effect size of the odds ratio for four categories of NCDs concerning gender differences through an analysis of historical data collected over 12 years. This was accomplished to present the whole phenomenal situation by evaluating the number of NCDs compared to the number of non-NCDs among individuals of each gender. Although gender is one of the risk factors for NCDs, as a nonmodifiable determinant of health, the causes of NCDs are multifactorial. Various determinants contributing to these NCDs are intriguing to find out and investigate, especially modifiable determinants of health. However, because of the limited data on any factors from the source of data that this study can gather, the multifaceted origins of contributing diseases may be limited for analysis in this current study. Therefore, to fulfill and demonstrate how the phenomenon goes on, it is suggested that future research needs to be conducted to explore in detail other dimensions related to contributing disease risks, such as how various aspects of illness are experienced, individual behaviors related to their health, access to and utilization of healthcare services, how to respond to treatment, and the overall impact on health. Additionally, multifactorial factors, such as socioeconomic status and health risk behaviors (e.g., smoking, alcohol and substance use, physical activity, dietary behaviors, and mental and emotional health), should be taken into consideration.

## Data Availability

The data on which this paper is based are available at the Strategy and Planning division of the Office of the Permanent Secretary Ministry of Public Health, at https://spd.moph.go.th/illness-report/. However, each annual dataset was presented in PDF, and Excel format. They are reports that solely provided content in Thai without full English translations.
